# Combined medial patellofemoral ligament and medial quadriceps tendon-femoral ligament reconstruction for patellar instability: a systematic review

**DOI:** 10.1186/s13018-025-06621-2

**Published:** 2026-01-10

**Authors:** Yi Hua, Hua Wang, Xu Yang

**Affiliations:** Department of Joint Surgery and Sports Medicine, Zhongnan Hospital of Wuhan University, Wuhan, 430071 China

**Keywords:** Patellar instability, Medial patellofemoral ligament, Medial quadriceps tendon-femoral ligament, Medial patellofemoral complex, Reconstruction

## Abstract

**Background:**

Data on the clinical outcomes of combined medial patellofemoral ligament (MPFL) and medial quadriceps tendon femoral ligament (MQTFL) reconstruction for patellar instability remain scarce.

**Methods:**

A systematic literature search was conducted in Web of Science, Embase, and PubMed on June 9, 2025. All studies reporting clinical outcomes after combined MPFL/MQTFL reconstruction for patellar instability were included. Methodological quality was assessed using the Methodological Index for Non-Randomized Studies (MINORS). General characteristics, surgical technique, clinical outcomes, satisfaction, and complications were recorded.

**Results:**

Five studies comprising 180 patients (188 knees) were included. The mean age was 14.8 years (range 12–22 years), and the mean follow-up was 34.6 months (range 24–49 months). Children and adolescents accounted for 67.8% of the cohort (*n* = 122). The mean Insall-Salvati ratio (ISR) or Caton-Deschamps index (CDI) was 1.2, and the mean tibial tubercle-trochlear groove (TT-TG) distance was 16.6 mm. Trochlear dysplasia was present in 86.2% of knees (162/180). Allografts were the most commonly used grafts (*n* = 103, 54.8%). Methods of quadriceps tendon fixation included soft tissue tunnel fixation (*n* = 61, 37.2%), and soft tissue suture fixation (*n* = 103, 62.8%). The overall complication rate was 8.0% (15/188), with recurrent dislocation occurring in 2.1%. No patellar fractures or growth distrubances were reported.

**Conclusions:**

Combined MPFL and MQTFL reconstruction is a safe and effective technique for treating patellar instability in both pediatric and adult patients, demonstrating low rates of recurrent dislocation and complications. However, the optimal surgical technique remains controversial.

## Introduction

Lateral patellar dislocation is a common knee injury, particularly in young, active individuals [1, 2]. The medial patellofemoral ligament (MPFL) is the primary static restraint to lateral patellar translation, providing approximately 50–60% of the restraining force [3–5]. MPFL rupture occurs in up to 90% of acute dislocations, and MPFL reconstruction has become the standard treatment for recurrent patellar instability [6–8].

Anatomical studies have shown that the proximal fibers of the MPFL extend to the quadriceps tendon, forming the medial quadriceps tendon-femoral ligament (MQTFL) [9, 10]. Tanaka et al. introduced the concept of the medial patellofemoral complex (MPFC), which comprises both the MPFL and MQTFL [11]. Biomechanical evidence suggests that the MQTFL contributes significantly to patellofemoral stability, particularly in full extension [12–14].

Although isolated MPFL reconstruction yields excellent outcomes and low redislocation rates [8, 15, 16], it remains unclear whether adding MQTFL reconstruction improves results. No consensus currently exists regarding indications or optimal surgical techniques for combined MPFL/MQTFL (MPFC) reconstruction [17, 18].

This systematic review aimed to evaluate the clinical outcomes of combined MPFL and MQTFL reconstruction for patellar instability. We hypothesized that this procedure would provide favorable outcomes with low complication rates.

## Methods

This systematic review was conducted in accordance with the Preferred Reporting Items for Systematic Reviews and Meta-Analyses (PRISMA ) guidelines [19].

### Literature research

On June 9, 2025, two authors (Y.H. and X.Y.) independently searched PubMed, Embase, and Web of Science using a comprehensive strategy.

The search strategy was as follows (PubMed for example): (((((((“Patellar Dislocation“[Mesh]) OR (Patellar Dislocation[Title/Abstract])) OR (patellar subluxation[Title/Abstract])) OR (patellar instability[Title/Abstract])) OR (Recurrent patellar dislocation[Title/Abstract])) OR (recurrent patellofemoral instability[Title/Abstract])) AND (((((Medial patellofemoral complex[Title/Abstract])) OR (MPFC[Title/Abstract])) OR (Medial patellofemoral complex reconstruction[Title/Abstract])) or (Medial quadriceps tendon femoral ligament and medial patellofemoral ligament [Title/Abstract])) OR (MQTFL and MPFL[Title/Abstract])).

### Study selection

Inclusion criteria: studies reporting clinical outcomes of combined MPFL and MQTFL reconstruction for patellar instability.

Exclusion criteria: reviews, conference abstracts, case reports, cadaveric/biomechanical studies, technique-only articles, non-English publications, and duplicate patient cohorts.

Titles, abstracts, and full texts were screened independently by two authors (Y.H. and X.Y.). Disagreements were resolved by the senior author (H.W.). Reference lists of included studies were hand-searched.

### Quality assessment

Methodological quality was evaluated using the Methodological Index for Non-randomized Studies (MINORS) tool [20]. Scores of 13–16 (non-comparative) or 21–23 (comparative) indicated low risk of bias.

### Data extraction

Data were extracted independently by two authors and included study characteristics, patient demographics, preoperative measurements, surgical details, patient-reported outcome measures (PROMs), return to sport, satisfaction, complications, and radiological outcomes.

### Statistical analysis

Due to heterogeneity among the included studies, the data could not be pooled for meta-analysis calculations. Therefore, descriptive analysis was used for numerical characteristics, including age, follow-up time, clinical and radiological outcomes. The degree of agreement for MINORS criteria was calculated using the Cohen κ coefficient. If the standard deviation was not reported, the authors were contacted to obtain it. Otherwise, the value was treated as unreported.

## Results

### Study selection

**Fig. 1:**
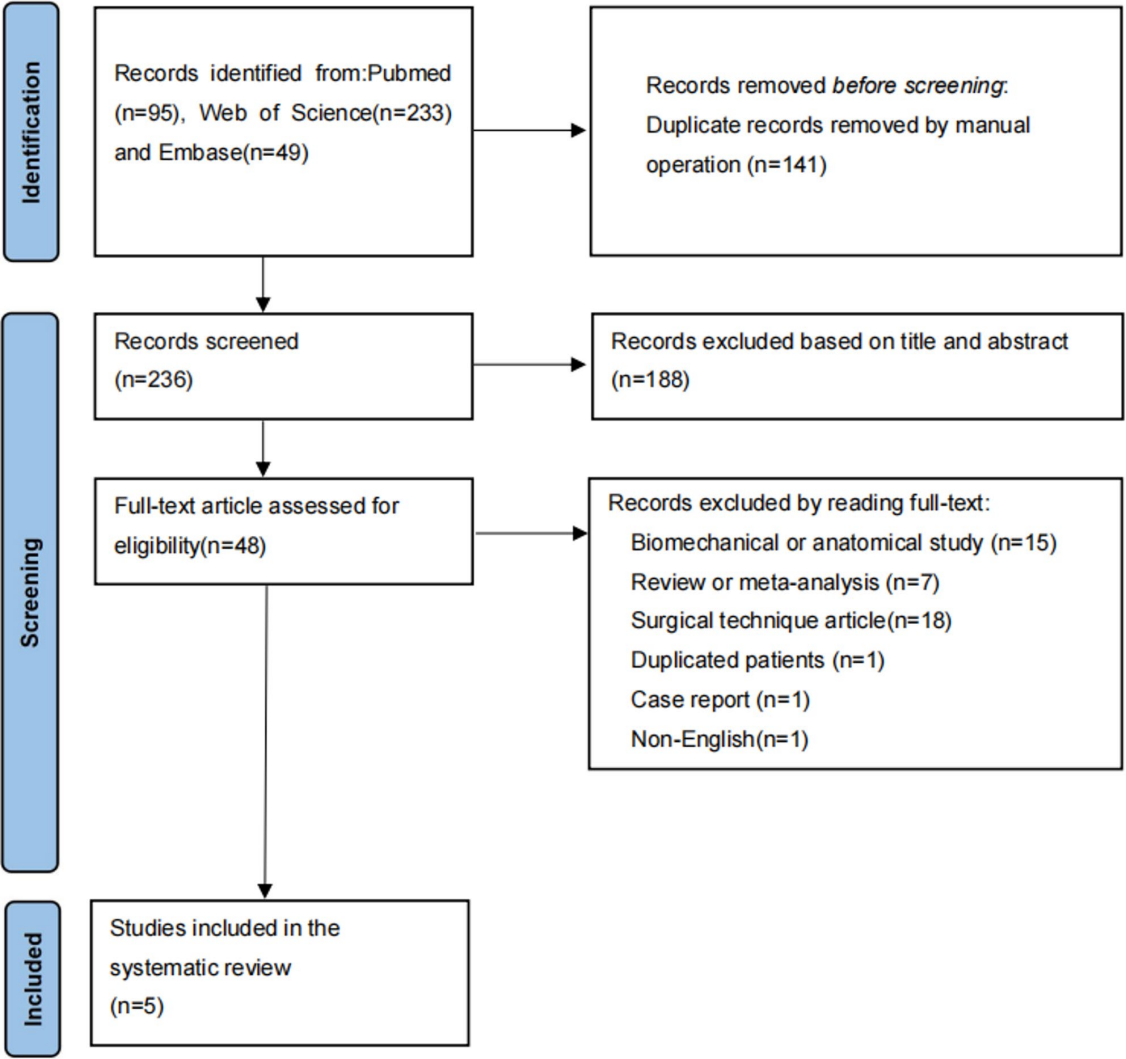
Article selection flowchart following the PRISMA guidelines

The search yielded 377 records. After removing duplicates and screening, five studies published between 2019 and 2025 were included (PRISMA flowchart-Figure 1) [21–25].

## Study characteristics

Three retrospective case series (Level IV) [21, 23, 25] and two retrospective cohort studies (One level IV [22], One level III [24] ) were included. Mean MINORS scores were 12 for the 3 non-comparative [21, 23, 25] and 21 for 2 comparative studies [22, 24]. Only one study [24] was classified as low risk of bias. Study characteristics are shown in Table 1.

**Table 1:** Study characteristics^*a*^

First author	Publication year	Journal	Study design	MINORS score	LOE
Spang [21]	2019	*J Pediatr Orthop*	Case series	12/16	4
Hu [22]	2024	*Arthroscopy*	Cohort study	20/24	4
Zein [23]	2024	*Orthop J Sports Med*	Case series	13/16	4
Reikersdorfer [24]	2025	*Arthroscopy*	Cohort study	22/24	3
Turazza [25]	2025	*Int Orthop*	Case series	11/16	4

^*a*^MINORS: Methodological Index for Non-randomized Studies; LOE: level of evidence

### Patient demographics

A total of 188 knees in 180 patients were analyzed. Mean age was 14.8 years (65% female). Mean follow-up was 34.6 months (range, 24–49 months) (Table 2). Children/adolescents comprised 67.8% of patients. Mean TT-TG distance was 16.6 mm, mean patellar height (CDI/ISR) was 1.2, and trochlear dysplasia was present in 86.2% of knees (Table 3). Four studies [21, 23–25] reported the outcomes of MPFC reconstruction in children and adolescents (*n* = 122, 67.8%), while one study [22] reported the outcomes in adults (*n* = 58, 32.2%). Among these, four studies [21–23, 25] focused on recurrent patellar dislocation (*n* = 128, 71.1%), and 1 study [24] involved first-time patellar dislocation (*n* = 52, 28.9%) (Table 4).

**Table 2:** Characteristics of included Patients^*a*^

First author	Patients, no.	Knees, no.	Mean age, y	Female/Male, no.	Mean follow-up, mo
Spang [21]	25	27	15	15/10	24
Hu [22]	58	61	22	37/24	49
Zein [23]	24	24	12	16/8	40
Reikersdorfer [24]	52	52	12	37/15	31
Turazza [25]	21	24	13	NR	29

^*a*^NR: not reported

**Table 3:** Preoperative patient measurements^*a*^

First author	CDI/ISR	Dejour classification	TT-TG distance, mm
Spang [21]	1.1 ± 0.2	A/B:22, C/D:4	17.2 ± 3.8
Hu [22]	1.2 ± 0.2	A:15, B:17, C:20, D:9	19.3 ± 3.4
Zein [23]	1.1 ± 0.1	A:9, B:6, C:4, D:2	14.7 ± 4.3
Reikersdorfer [24]	NR	A:21, BCD:9	NR
Turazza [25]	1.3 ± 0.2	A:9, B:4, C:10, D:1	15.3 ± 3.6

^*a*^ISR: Insall-Salvati ratio; CDI: Caton-Deschamps index; TT-TG: tibial tubercle-trochlear groove

**Table 4:** Surgical details^*a*^

First author	Population	Dislocation type	Graft type	Femoral fixation	Patellar fixation	Quadriceps fixation
Spang [21]	C and Ado	Recurrent	Allograft	Suture anchor	Suture anchor	Suture
Hu [22]	Adu	Recurrent	SemiT	Bone tunnel	Bone tunnel	Soft tunnel
Zein [23]	C and Ado	Recurrent	QT	Pulley	Suture	Suture
Reikersdorfer [24]	C	First-Time	Allograft	Suture anchor	Suture anchor	Suture
Turazza [25]	C and Ado	Recurrent	Allograft	Bone tunnel	Suture anchor	NR

^*a*^C: children; Ado: adolescent; Adu: adult; Allo: allografts; Recurrent: recurrent patellar dislocation; First-Time: first-time patellar dislocation; Gra: gracilis; SemiT: semitendinosus; QT: quadriceps tendon; Pulley: soft tissue pulley

### Surgical techniques

#### Graft choice

Allografts were used in 54.8% of cases [21, 24, 25]. Autograft was used in two studies [22, 23], including semitendinosus tendon (*n* = 61, 32.4%) [22], and quadriceps tendon (*n* = 24, 12.8%) [23].

#### Patellar fixation

The methods of patellar fixation included suture anchor fixation (*n* = 103, 54.8%) [21, 24, 25], bone tunnel fixation (*n* = 61, 32.4%) [22], and soft tissue suture fixation (*n* = 24, 12.8%) [23].

#### Quadriceps tendon fixation

The methods of quadriceps tendon fixation included soft tissue tunnel fixation (*n* = 61, 37.2%) [22], and soft tissue suture fixation (*n* = 103, 62.8%) [21, 23, 24].

#### Femoral fixation

The methods of femoral fixation included bone tunnel fixation with interference screw (*n* = 85, 45.2%) [22, 25], suture anchor fixation (*n* = 79, 42.0%) [21, 24], and soft tissue pulley fixation around the adductor magnus tendon (*n* = 24, 12.8%) [23]. The femoral attachment sites included distal to the physis in skeletally immature patients (*n* = 103, 54.8%) [21, 24, 25], the Schöttle point in adults (*n* = 61, 32.4%) [22], and the adductor magnus tendon (*n* = 24, 12.8%) [23]. The surgical details of combined MPFL and MQTFL reconstruction are presented in Table 4.

### Subjective clinical outcomes

The most frequently reported patient-reported outcome measures (PROMs) are summarized in Table 5, including Tegner score (2 studies) [22, 24], Kujala score (5 studies) [21–25], Lysholm score (3 studies) [21, 22, 25], and IKDC score (4 studies) [21, 22, 24, 25]. All studies reporting pre- and postoperative scores showed significant improvement in Kujala, Lysholm, IKDC, and Tegner scores. Notably, only 2 studies [22, 23] provided preoperative PROM data, and both showed significant improvement from pre- to postoperative assessment. Patellar tilt angle (PTA) improved markedly in the two studies reporting this parameter [22, 23]. Return-to-sports (RTS) rates were reported in 3 studies [21, 22, 24], ranging from 77% to 90% (Table 6). Patient subjective satisfaction was assessed in 1 study (9.2) [22].

### Complications

The overall complication rate was 8.0% (15/188 knees) (Table 6). Recurrent dislocation occurred in 2.1%, subluxation in 1.1%, stiffness/arthrofibrosis in 2.2%, and quadriceps weakness in 1.1%. No patellar fracture or growth plate disturbances were reported.

**Table 5:** Patient reported outcome measures^*a*^

First author	Tegner		Kujala		Lysholm		IKDC	
	Pre	Post	Pre	Post	Pre	Post	Pre	Post
Spang [21]	NR		NR	85.9 ± 13.9	NR	84.3 ± 13.5	NR	81.5 ± 15.2
Hu [22]	3.1 ± 1.4	4.8 ± 1.5	58.3 ± 15.8	93.1 ± 5.6	63.0 ± 15.1	93.8 ± 6.5	55.3 ± 15.6	87.1 ± 7.9
Zein [23]	NR		59.1 ± 15.3	93.6 ± 3.5	NR		NR	
Reikersdorfer [24]	NR	6.0 ± 1.4	NR	91.5 ± 7.8	NR		NR	85.5 ± 12.1
Turazza [25]	NR		NR	92.8 ± 7.5	NR	94.3 ± 6.3	NR	91.2 ± 7.2

^*a*^Data are reported as mean ± SD; Pre: preoperative; Post: postoperative; IKDC: International knee documentation committee; NR: not reported

**Table 6:** Satisfaction rate, RTS, complication, and radiological assessments

First author	Satisfaction	RTS	Complication	Radiological assessments
Spang [21]	NR	77%	Redislocation (n = 2)	NR
Hu [22]	9.2	90%	Redislocation (n = 2); Stiffness(n = 1)	PTA
Zein [23]	NR	NR	Stiffness(n = 1); Quadriceps weakness(n = 1)	PTA
Reikersdorfer [24]	NR	89%	Arthrofibrosis(n = 2); Quadriceps weakness(n = 1); Superficial infection(n = 1); Local allergic reaction (n = 1); Contact dermatitis (n = 1)	NR
Turazza [25]	NR	NR	Subluxation(n = 2)	NR

^*a*^NR: not reported; RTS: return to sports; PTA: patellar tilt angle

## Discussion

This systematic review demonstrates that combined MPFL and MQTFL (MPFC) reconstruction is safe and effective for patellar instability in both skeletally immature and mature patients, with a re-dislocation rate of only 2.1% and no growth-plate injuries or patellar fractures.

Over the past two decades, MPFL reconstruction has become a mainstream procedure for the treatment of patellar dislocation due to favorable clinical outcomes and low rates of redislocations [26, 27]. Although multiple MPFL reconstruction techniques have been described, the core concept of MPFL reconstruction remains the optimal restoration of the native anatomy [28]. The term MPFC was first proposed by Tanaka [11] to describe the static medial stabilizer of the patella, which typically includes the MPFL and MQTFL. The MQTFL acts as a continuation of the MPFL, inserts anteriorly into the distal quadriceps tendon, and may play an important role in maintaining patellofemoral joint stability [29]. The MPFL is the primary static stabilizer, providing about 50–60% of the restraining force against lateral translation of the patella during the first 30° of knee flexion [30]. However, the MQTFL may function as a dynamic stabilizer, which plays an important role in resisting lateral patellar translation during full knee extension [31, 32]. A biomechanical study by Spang et al. [33] found that both MQTFL and MPFL reconstructions restored patellofemoral stability, with MQTFL reconstruction more closely restoring native resistance to lateral patellar translation. Bowman et al. [34] reported that MQTFL reconstruction is a safe and effective procedure for treating patellar instability, without the risk of patella fracture compared to MPFL reconstruction. A matched-cohort study by Shankar et al. [35] found that there were no significant differences in knee pain, satisfaction, functional outcome scores, or rates of redislocation between MQTFL and MPFL reconstruction.

Currently, there is still debate about whether to combine MQTFL and MPFL reconstruction in the treatment of patellar instability, however, the most anatomic repair may involve both the MQTFL and the MPFL [17, 36]. A biomechanical study by White et al. [13] demonstrated no significant differences in lateral patellar translation, patellar tilt, contact areas, and contact forces among isolated MQTFL reconstruction, isolated MPFL reconstruction, and combined MPFL/MQTFL (MPFC) reconstruction. Similarly, Dahm et al. [14] found no significant differences in patellofemoral contact pressure and lateral patellar translation across the three techniques; however, isolated MQTFL and combined MPFL/MQTFL (MPFC) reconstruction more closely resembled the intact knee state. Hu et al. [22] compared the clinical and radiographic outcomes of MPFL and MPFC reconstruction for RPD. It was demonstrated that both surgical procedures yielded similar outcomes. In the present systematic review, MPFC reconstruction resulted in favorable clinical outcomes in both skeletally immature and mature patients, with a low re-dislocation rate of 2.1%.

Isolated MPFL reconstruction has demonstrated satisfactory clinical and radiographic outcomes in adults with patellar instability; whether to combine MPFL/MQTFL (MPFC) reconstruction in adults remains debatable [8, 37]. Current evidence suggests no additional benefit of MPFC over MPFL reconstruction for treating patellar dislocation in adults [22]. Currently, most scholars recommend combined MPFL/MQTFL (MPFC) anatomical reconstruction for patellar instability in patients with open growth plates, as it may be associated with better knee function and return to sports [23, 24, 38, 39]. In skeletally immature patients, combined reconstruction using soft-tissue or physeal-sparing techniques appears particularly attractive because it avoids patellar bone tunnels (reducing fracture risk) while providing robust medial restraint. In adults, the additional benefit of MQTFL reconstruction remains uncertain.

 However, in patients with patella alta, excessive proximal MQTFL tension may increase failure risk or cause medial overconstraint [40]. This is because the proximal and distal bundles of the MPFC are anisometric, providing differential resistance to lateral patellar translation at varying flexion angles, and patella alta requires greater knee flexion before the patella engages the trochlea [32, 41]. Hu et al. [22] reported that MPFC reconstruction may not provide additional benefits for patients with patella alta. In addition, compared to MPFL reconstruction, MPFC reconstruction increases the area of the fan-shaped structural tissue, which may lead to medial over-constraint of the patellofemoral joint and result in a series of complications [21, 23]. Consequently, when performing MPFC reconstruction in patients with patella alta, surgeons should pay special attention to proximal MQTFL reconstruction and carefully select the most appropriate graft fixation position and technique.

The absence of a standardized technique reflects ongoing evolution in understanding of the MPFC. Graft choice, femoral attachment (anatomic vs. physeal-sparing), and fixation methods vary widely. Particularly for patients with open physes, most scholars tend to adopt soft tissue fixation techniques for MPFC reconstruction [23, 42–44]. Previously, when performing MPFL reconstruction in skeletally immature patients, soft tissue fixation techniques were usually adopted to avoid injury to the physis [45, 46]. With advances in patellofemoral joint anatomy and biomechanical research, surgeons have shifted toward anatomical reconstruction protocols, establishing the femoral tunnel distal to the physis and using bony fixation for MPFL reconstruction [47]. Compared to non-anatomical MPFL reconstruction, this anatomical approach with lower re-dislocation rates and higher return-to-sport rates [45]. However, there have been no reported cases of using anatomical MPFC reconstruction to treat patellar dislocation in patients with open growth plates. Anatomical MPFC reconstruction may become a mainstream surgical approach in the future. Similar to MPFL reconstruction, it can be combined with bony procedures to treat patellar instability.

### Limitations

There are several limitations to this study. Only five studies, mostly retrospective and with small sample sizes, were available for analysis. Heterogeneity precluded meta-analysis. Higher-level prospective studies with longer follow-up are needed.

## Conclusion

Combined MPFL and MQTFL reconstruction is a safe and effective treatment for patellar instability in children, adolescents, and adults, offering low re-dislocation and complication rates. Although no standardized technique currently exists, individualized anatomical reconstruction of the MPFC represents an important future direction.

## Data Availability

No datasets were generated or analysed during the current study.

## Abbreviations

MPFC: Medial patellofemoral complex
MQTFL: Medial quadriceps tendon femoral ligament
MPFL: Medial patellofemoral ligament
TT-TG: Tibial tuberosity-trochlear groove
MINORS: Methodological index for non-randomized studies
CDI: Caton-Deschamps index
ISR: Insall-Salvati ratio
